# Salt consumption and the risk of chronic diseases among Chinese adults in Ningbo city

**DOI:** 10.1186/s12937-020-0521-8

**Published:** 2020-01-29

**Authors:** Yi Lin, Qiuhong Mei, Xujun Qian, Tianfeng He

**Affiliations:** 1 Center for Health Economics, School of Economics, Faculty of Humanities and Social Sciences, University of Nottingham, Ningbo China, 199 Taikang East Road, University Park, Ningbo, 315100 China; 2 Department of Health and Education, Ningbo Municipal Center for Disease Control and Prevention, 237 Yongfeng Road, Ningbo, 315010 China; 3 Departmentof Health and Management, Ningbo First Hospital, 59 Liuting Street, Ningbo, 315010 China

**Keywords:** Salt intake, Nutrition and health survey, Chronic diseases, Obesity, Diabetes, Hypertension, Chinese

## Abstract

**Background:**

Chronic diseases have become one of essential public health concerns, leading causes of mortality in China. It is related to the changes in dietary pattern and dietary behavior. The objectives are to assess daily salt intake in Chinese people living in Ningbo and to examine its relationship with health outcomes.

**Methods:**

Our study used data from health and nutrition survey in 2017. This study included 2811 adults aged 18–79 years (48% males) from urban and rural areas in Ningbo. A food frequency questionnaire together with demographic, physical and medical questionnaires was used to collect dietary intake, demographic, lifestyle and medical information. Ordinal logistic regression was used in the statistical analysis.

**Results:**

The mean daily salt intake (13.0 g/day) of the participants was higher than the Chinese dietary reference intake (DRI, 6 g/d), which was related to higher risk of pre-hypertension and hypertension. Stratified by gender, education and lifestyle factors, daily salt intake was only significant in the blood pressure category (male: *P* = 0.048; less education: *P* = 0.003; urban: *P* = 0.006; no regular physical activity: *P* = 0.005, no regular smoking: P = 0.006). Ordinal logistic regression model shows that daily salt intake was significantly associated with higher odds of developing hypertension.

**Conclusion:**

The daily salt intake of the majority of citizens living in Ningbo exceeded Chinese DRI and may increase the risk of hypertension. Moreover, public health intervention of salt restriction is necessarily needed for the prevention and control the ongoing epidemic of chronic diseases.

## Introduction

During past 3–4 decades, China has been facing an epidemiological shift in morbidity and mortality from under-nutrition related infectious diseases to over-nutrition related non-communicable diseases (NCD) due to rapid population ageing, urbanization and lifestyle changes [1]. National Health and Family Planning Commission of the People’s Republic of China reported that the prevalence of obesity, hypertension and diabetes among Chinese adults at 18 years old and above in 2012 were 30.1%, 25.2% and 9.7%, respectively [2]. In China, about 87% of all-cause mortality in 2012 and 77% of total disability-adjusted life-year loss in 2010 were attributed to NCDs [2, 3].

Nutrition and dietary patterns have been the major modifiable determinants of chronic diseases [4–6]. Significant evidence has proved that human’s diet has strong impact on health [5–8]. With increasing consumption of energy-dense foods in China, overconsumption of salt has been identified as a risk factor for development of major chronic diseases [9, 10], although salt/sodium not only is an important preservative method [11], but also makes essential contribution to eating pleasure and satisfaction [12]. Due to an important flavor, salt/sodium can determine the changes of dietary behavior, food choices and food preference [12]. Previous studies showed that high consumption of salt is positively associated with obesity having great body mass index (BMI) and waist circumference, hypertension, diabetes, cardiovascular diseases [13, 14].

For the past decades, salt intake has been increased gradually, exceeding the recommended daily intake (5 g/d) proposed by world health organization (WHO) [15]. Results from the Chinese nutrition and health surveillance in 31 provinces with a 3-day food weighed record [16] and the China nutrition and transition cohort study covering 15 provinces using a standard three consecutive 24-h retrospective method [17] showed that the nationwide salt intake is 9.6 g/d in 2010–2012 and 9.9 g/d (sodium: 3960.0 mg/d) in 2015, respectively. Both studies reported that men consumed higher salt than women, middle aged group in particular [17]. A recent study showed that salt intake was 8.9 g/d in 2015 in Zhejiang province [18].

Information on salt intake in Ningbo is not well studied and updated. Therefore, the aims of the current study are (1) to estimate the intake levels of salt in a representative sample of Ningbo, China and (2) to examine the association between salt intake and risk factors of health outcome including the status of BMI, fasting blood glucose and blood pressure.

## Methodology

### Study design and study sample

This current study is the baseline study of a local cohort survey conducted in Ningbo in 2017. A multistage, random cluster sampling procedure was used to draw the target samples, covering socio-economic status, public resources and health aspects in both urban and rural areas. A weighting method was carried out further based on the method of the sixth Ningbo census survey in 2010 from Ningbo Bureau of Statistics, following a national standardized sampling procedure proposed by National Bureau of Statistics of China [19]. The weighing sampling procedure considering the characteristics of urban-rural areas was to ensure a representativeness of socio-economic status. The urban and rural areas were categorized based on the definition proposed by the government of Ningbo. The counties of Cixi, Yuyao, Ninghai and Xiangshan are categorized into rural areas and the rest regions (Beilun, Fenghua, Jiangbei, Haishu, Yinzhou, and Zhenhai) are urban areas. City, town, county and village were stratified by income (low, middle, and high) in Ningbo. Four street blocks or counties were selected at each city/town level as primary sampling units. Two neighborhood committees or villages were selected as secondary sampling units at each city, town and county level. Approximately 120 out of 750 household families from each residential region were randomly selected in each neighborhood committee or village. KISH method, which is a way of randomly choosing target respondents for a household survey, was used to select one family member aged 15 to 80 years at the final sampling procedure, until 100 family members in each residential region were interviewed and recruited in this survey [20]. In total, 4973 individuals participated in the baseline study.

In the current study, 2963 out of 4973 recruited local citizens aged 18 to 79 years, who were not pregnant or lactating during data collection, not suffering from mental, physical diseases, severe chronic diseases, completing a food frequency questionnaire (FFQ) together with additional questionnaires including demography, lifestyle and self-health check report were included for the final data analysis.

The current study was conducted according to the guidelines proposed by Ningbo Municipal Center for Disease Control and Prevention (CDC) and all procedures involving human subjects were approved by Institutional Review Board of Ningbo Municipal CDC [No. IRB 201603]. Written and verbal informed consent was obtained from all participants.

### Dietary intake assessment

A standard FFQ was developed based on the guidelines proposed by the China CDC and Ningbo CDC [21]. This FFQ was used to collect participants’ quantitative dietary information on usual foods and dietary behavior. Dietary information was collected through 5 sections including family dietary background, family dietary behavior on seasoning, personal dietary behavior, alcohol consumption and water consumption. The section of family dietary background focused on how many family members usually had daily meals (breakfast, lunch and dinner) at home for the past month. Information of personal dietary behavior was on how often participants ate at home and outside for the past week. Personal dietary behavior for the past month was about the frequency of usual foods consumed (fresh vegetables, legumes and legume products, fresh fruit, red meat, poultry and shrimp, milk and milk products, eggs and egg products, barbeque and pickles). The section of family dietary behavior on seasoning focused on the information related to the amount of seasoning (salt, soya source, eatable oil and MSG) consumed for the past month. Dietary information of seasoning consumption was collected through 2 questions [How many packages/bottles did you eat for the past month? What was the quantity of one package/bottle for those consumed seasoning (g or litre)].The section of alcohol consumption was about when participants started drinking behavior, how many litres/bottles and types of alcohol they drank for the past week. The section of water consumption was about whether participants drank water/tea/coffee, how much (200 ml as the unit of a glass) and how often they drank for the past week. All the participants were interviewed by well-trained social healthcare workers from local health centers/hospitals and researchers from Ningbo CDC. The misreporting of dietary information has been correlated and excluded by experienced researchers during the field visit and statistical method. For example, salt intakes extremely higher than the daily reasonable consumption were excluded in the current study.

### Anthropometric measurements

Weight (kg) and height (m) were self-reported to the 0.1 kg and the 0.1 cm, respectively. Thereafter, BMI was calculated as weight (kg)/height (m^2^). Participants were classified into four BMI categories according to China Obesity Task Force as follow: underweight (< 18.5 kg/m^2^), normal weight (18.5–23.9 kg/m^2^), overweight (24.0–27.9 kg/m^2^), and obesity (≥28.0 kg/m^2^) [22].

### Biomarker measurements

Biomarker information including blood pressure and fasting blood glucose was reported by healthcare workers at local health centers and hospitals. Blood pressure was measured at the upper limb brachial artery in a seated posture by an electronic blood pressure meter after participants were asked to rest for 5 mins. Blood pressure was measured 3 times at 30-second intervals. Therefore, the average blood pressure was calculated based on these 3 measurements. Participants, who were willing to be involved in the blood sampling, were asked to fast after 8 pm on the previous day. In addition, medical questionnaire, including fasting status, acute infection, allergies, smoking, family medical history and medication, was completed by the participants for assessing blood pressure.

Systolic blood pressure (SBP) and diastolic blood pressure (DBP) were reported to the 0.1mmHG and blood glucose was to the 0.1 mmol/L. Blood pressure and fasting blood glucose were measured following the instruction of Chinese national guidelines for the prevention and control of hypertension in primary care [23] and national guidelines for the prevention and control of type 2 diabetes in primary care [24], respectively.

Blood pressure was defined as 4 following categories: low blood pressure (70 ≤ SBP < 90 and 40 ≤ DBP < 80), normal blood pressure (90 ≤ SBP < 120 and 60 ≤ DBP < 80), high blood pressure /pre-hypertension (120 ≤ SBP < 140 and/or80 ≤ DBP < 90) and hypertension (≥140) [25]. Fasting blood glucose was categorized into 3 groups: low level (< 4.0), normal level (4.0–6.9) and high level/diabetes (≥7.0) [24].

### Level of education

Participants were asked to report the highest degree that they had obtained during the interview. Three categories of education level were created: (1) no education or lower secondary education; (2) secondary education; (3) higher education (bachelor, master or above).

### Statistical analysis

The percentage and mean intake with standard error among risk groups were presented as descriptive analysis. Based on Central Limit Theorem, normal distribution was applied to daily salt intake across factor groups and health outcomes. Equality of the variances was examined using Levene’s test. Statistical differences of the prevalence among the groups of dietary reference intake (DRI) and health outcomes were assessed using Z test. Student’s *T*-test and ANOVA with Bonferroni correction/ Games-Howell were used to examine the difference of mean intakes within risk groups.

A two-tailed partial correlation was used to examine the correlation between daily salt intake and health outcomes (the status of BMI, fasting blood glucose and blood pressure), after adjusting for confounding factors (gender, age and education level) and lifestyle factors (regular smoking longer than 6 months, regular alcohol drinking longer than 6 months and regular physical activity). Ordinal logistic regression was fitted to investigate the association of salt intake (continuous variable) and health outcomes through three models:(1) unadjusted model; (2) model adjusted for gender, age and education level; (3) model further adjusted for lifestyle factors based on Model 2. Two-way interactions were examined between independent variable and confounding factors, and lifestyle factors in Model 3 as well. Interactions were only maintained in Model 3 if they were statistically significant. Statistical power was examined and higher than 80%.

Results were considered statistically significant at a two-tailed level of 0.05. Statistical analyses were conducted using the STATA statistical software package version 15 (2017).

## Results

### Study population

In total, 2811 (48% men) out of 4973 recruited individuals participated in the survey and completed a FFQ with valid demographic, lifestyle and medical information. Among all 2963 individuals who completed the FFQ and other relevant questionnaires, 4 individuals had invalid BMI, 57 individuals got misreported fasting blood glucose, 48 individuals had extremely low/high SBP and 59 individuals had extremely low SBP.

Approximately 40% participants were living in rural areas. Around 21% participants were in the group with higher education, young adult (18–40 years) in particular (Table 1). 87% participants had medium monthly household income (6000–180,000/year). Almost half participants had regular physical activity, while the majority reported healthy lifestyles with no regular smoking (78%) and no regular alcohol drinking (80%). Considering health outcomes of chronic diseases, 26.8% and 5.4% participants were defined as overweight and obesity, respectively. Only 4% individuals were reported to have high fasting blood glucose status and tend to develop type 2 diabetes. On the contrary, 73% individuals were considered to have pre-hypertension, the middle age ones (41–65 years, 65%) in particular.

**Table 1:** Socio demographic characteristics of Chinese citizen living in Ningbo municipal center stratified by age

Socio demographic characteristic	Total (n)	Age (%)		
		18–40	41–65	> 65
Sex				
Male	1354	28	64	9
Female	1457	30	61	9
Ethic group				
Han	2799	29	62	9
Geography				
Urban	1687	30	60	10
Rural	1124	27	66	7
Education				
No education or lower secondary	1769	11	77	13
Secondary	462	41	55	3
Higher education	580	76	24	1
Occupation				
Public service (e.g. teachers, administers at governments)	356	58	40	2
Laborers (e.g. workers, famers)	1260	16	73	11
Students	38	92	8	0
Other sectors (e.g. business)	482	46	51	3
Others	675	22	66	12
Household Income				
Low (< 6000/y)	24	17	58	25
Medium (6000–180,000/y)	2441	26	64	9
High (≥ 180,000/y)	346	48	49	4
Regular Physical Activity				
Yes	1402	27	63	10
No	1410	31	62	7
Regular Smoking				
Yes	611	22	70	8
No	2200	31	60	9
Regular Alcohol Drinking				
Yes	570	16	75	10
No	2241	32	59	8
BMI				
Underweight	205	60	34	6
Normal Weight	1699	30	62	8
Overweight	754	20	70	10
Obesity	153	24	64	12
Fasting blood glucose				
Low	80	39	55	6
Normal	2616	30	62	8
High/Diabetes	115	7	77	17
Blood pressure				
Low	4	25	75	0
Normal	417	53	45	3
Pre-hypertension	2057	27	65	8
Hypertension	333	10	71	20

### Daily salt intake and risk factors among health outcomes

The mean salt intake was 13.0 g/d (median: 10 g/d) among all the participants. Men consumed almost the same daily salt intake as women did (men: 13.0 g/d; women: 12.9 g/d). Considering the age factor, senior generation (> 65 years, 14.4 g/d) consumed significantly more salt compared to young adults (12.0 g/d) (*P* = 0.003). Additionally, rural participants (13.6 g/d) had significantly more daily salt intake compared to urban ones (12.5 g/d) (*P* = 0.027).

Daily salt intake was much higher than Chinese RDI (≥6 g/d) (84% participants, 82% men and 85% women). Participants exceeding Chinese DRI were observed to have significantly higher prevalence of overweight/obesity, high fasting blood glucose status, pre-hypertension among all groups of gender, age and residential areas as compared to those counterparts (Table 2). Additionally, the prevalence of daily salt intake exceeding Chinese DRI (≥6 g/d) was significantly higher than the prevalence of daily salt intake less than Chinese DRI across all the groups of gender, age and residential areas.

**Table 2:** Prevalence (%) of health outcomes in different risk groups by Chinese recommended daily intake

Dietary reference intake	Normal Weight	Overweight/obesity	Normal Fasting Blood Glucose	High Fasting Blood Glucose/Diabetes	Normal Blood Pressure	Pre-hypertension	Hypertension
Total							
< 6 g/d	10	5^b^	15	1^b^	3	12^d^	1^fh^
≥ 6 g/d	50^j^	24^bj^	78^j^	3^bj^	42^j^	61^dj^	11^fhj^
Male							
< 6 g/d	10	7^b^	16	1^b^	2	14^d^	2^fh^
≥ 6 g/d	48^j^	30^bj^	77^j^	3^bj^	8^j^	36^dj^	11^fhj^
Female							
< 6 g/d	10	3^b^	14	0^b^	4	10^d^	1^fh^
≥ 6 g/d	53^j^	25^bj^	79^j^	4^bi^	15^j^	60^dj^	10^fhj^
18–40							
< 6 g/d	11	5^b^	18	0	5	13^d^	0^eg^
≥ 6 g/d	52^j^	18^bj^	78^j^	1^bj^	22^j^	56^dj^	4^fh^
41–65							
< 6 g/d	10	5^b^	14	1^b^	2	12^d^	1^fh^
≥ 6 g/d	50^j^	31^bj^	78^j^	0^b^	9^j^	64^dj^	12^fhj^
> 65							
< 6 g/d	9	5^b^	15	1^a^	1	12^d^	2^fg^
≥ 6 g/d	47^j^	33^bj^	78^j^	7^b^	4	57^dj^	24^fhi^
Urban							
< 6 g/d	13	6^b^	11	1^b^	4	16^d^	1^fh^
≥ 6 g/d	50^j^	25^bj^	81^j^	4^bj^	11^j^	60^dj^	9^fhj^
Rural							
< 6 g/d	5	4^b^	21	1^b^	2	7^d^	1^fh^
≥ 6 g/d	53^j^	31^bj^	73^j^	3^bi^	14^j^	63^dj^	13^fhj^

^a^Significant difference across categories of weight and blood glucose, *P* < 0.05

^b^Significant difference across categories of weight and blood glucose, *P* ≤ 0.001

^c^Significant difference between normal blood pressure and pre-hypertension, *P* < 0.05

^d^Significant difference between normal blood pressure and pre-hypertension, *P* ≤ 0.001

^e^Significant difference between normal blood pressure and hypertension, *P* < 0.05

^f^Significant difference between normal blood pressure and hypertension, *P* ≤ 0.001

^g^Significant difference between pre-hypertension and hypertension, *P* < 0.05

^i^Significant difference between groups of Chinese dietary reference intake, *P* < 0.05

^j^Significant difference between groups of Chinese dietary reference intake, *P* ≤ 0.001

Table 3 shows that daily salt intake was significantly higher with increasing blood pressure status among all the participants (*P* = 0.006). Stratified by confounding factors, increasing daily salt intake was significantly associated with blood pressure status men (*P* = 0.048), urban areas (P = 0.006) and less educated group (*P* = 0.003). Daily salt intake among higher educated individuals deceased with increasing blood pressure status, although no significance was found. In terms of lifestyle factors, on one hand, higher daily salt intake was significantly associated with blood pressure status (*P* = 0005) among the group of no regular physical activity; on the other hand, higher daily salt intake was significantly associated with blood pressure status among healthy lifestyle including no regular smoking (*P* = 0.005) and alcohol drinking (*P* = 0.006).

**Table 3:** Mean daily salt intake^*^among risk groups by socio-demographic and lifestyle factors

Characteristics	BMI		Fasting Blood Glucose		Blood Pressure			*P*-value		
	Normal	Overweight/obesity	Normal	Diabetes	Normal	Pre-hypertension	Hypertension	BMI	Blood Glucose	Blood Pressure
Total	13.0 (0.330)	12.9 (0.386)	13.0 (0.254)	13.3 (1.2)	12.4 (0.538)	12.9 (0.265)	15.1 (1.0)	0.879	0.880	0.006
Sex										
Male	13.3 (0.537)	12.4 (0.505)	13.0 (0.395)	13.3 (1.5)	12.6 (1.1)	12.7 (0.381)	15.4 (1.6)	0.276	0.975	0.048
Female	12.7 (0.403)	13.5 (0.594)	12.9 (0.324)	13.3 (1.7)	12.3 (0.600)	12.8 (0.369)	14.7 (1.2)	0.291	0.845	0.129
Geography										
Urban	12.6 (0.463)	12.4 (0.556)	12.7 (0.246)	14.4 (1.7)	11.5 (0.735)	12.3 (0.359)	15.7 (1.8)	0.788	0.259	0.006
Rural	13.6 (0.433)	13.6 (0.511)	13.3 (0.515)	11.4 (2.4)	13.8 (0.763)^a^	13.5 (0.380)^a^	14.4 (0.894)	0.959	0.486	0.569
Education										
No education or lower secondary	13.5 (0.465)	13.5 (0.464)	13.5 (0.346)	13.9 (1.4)	11.9 (0.590)	13.3 (0.351)	16.0 (1.3)	0.905	0.883	0.003
Secondary	12.7 (0.746)	12.2 (1.1)	12.6 (0.611)	11.4 (1.6)	13.9 (1.8)	12.4 (0.650)	12.4 (1.6)	0.735	0.779	0.628
Higher education	11.6 (0.501)	10.8 (0.826)^a^	11.5 (0.412)	9.4 (3.2)^a^	12.2 (0.893)	11.3 (0.461)^a^	9.2 (1.1)^b^	0.414	0.487	0.264
Regular Physical Activity										
Yes	12.2 (0.400)	12.1 (0.513)	12.4 (0.322)	11.6 (0.805)	12.4 (0.790)	12.1 (0.343)	13.5 (0.993)	0.860	0.592	0.288
No	13.7 (0.524)^a^	13.7 (0.575)^a^	13.6 (0.392)^a^	15.6 (2.5)	12.4 (0.736)^a^	13.4 (0.402)^a^	17.2 (2.0)	0.971	0.402	0.005
Regular Smoking										
Yes	13.9 (0.632)	12.5 (0.676)	13.4 (0.473)	11.7 (1.4)	12.5 (1.3)	13.3 (0.528)	13.9 (1.1)	0.140	0.442	0.794
No	12.7 (0.382)	13.0 (0.464)	12.9 (0.287)	13.8 (1.5)	12.4 (0.585)	12.6 (0.306)	15.5 (1.3)	0.638	0.597	0.005
Regular Alcohol Drinking										
Yes	14.0 (0.651)	13.0 (0.695)	13.5 (0.488)	12.0 (1.7)	12.1 (1.3)	13.5 (0.551)	14.1 (1.1)	0.297	0.446	0.623
No	12.7 (0.379)	12.9 (0.457)	12. 8(0.293)	13.7 (1.3)	12.4 (0.583)	12.6 (0.302)	15.4 (1.3)	0.816	0.633	0.006

Values are mean intake with standard error

^a^Mean salt value was significantly different between categorical groups, *P* < 0.05

^b^Mean salt value was significantly different between categorical groups, *P* ≤ 0.001

### Association between daily salt intake and health outcomes among risk groups

The results of partial correlation show that daily salt intake was only associated with SBP and DBP (*P* = 0.043 and *P* = 0.008, respectively) (Table 4). The associations between health outcomes and daily salt intake were further assessed using ordinal logistic regression (Table 5). Significant associations between daily salt intake and blood pressure status were found in Model 1 (*P* = 0.014) and Model 3 (*P* = 0.044) after controlling for gender, age, education and lifestyle factors. No significant association was observed in Model 3. Interactions were not significantly in the models and removed from Model 3. The result in Model 3 indicates that increasing 1 g/d daily salt intake would result in a 1.006 times odds of getting higher blood pressure status, after adjusting for the potential confounding factors and lifestyle factors.

**Table 4:** Partial correlation of the relationship between daily salt intake and health outcomes at baseline study

Health Outcomes^a^	Daily salt intake	
	r	*P*-value
BMI	−0.009	0.642
Fasting Blood Glucose	−0.008	0.680
Systolic blood pressure	0.038	0.043
Diastolic blood pressure	0.050	0.008

^a^Controlling for gender, age, education, geography, physical activity, smoking and drinking

**Table 5:** Ordinal regression analysis of the association between the status of blood pressure and daily salt intake among Chinese participants living in Ningbo city

Salt intake^a^	OR^c^	P-value	95% CI	
			Lower Bound	Upper Bound
Model 1^b^	1.009	0.014	1.002	1.016
Model 2^b^	1.006	0.064	1.000	1.012
Model 3^b^	1.006	0.044	1.000	1.013

OR odds ratio; CI: confidence interval

^a^Hypertension reference category in the model

^b^Model 1: Unadjusted; Model 2, adjusted for gender, age and education level; Model 3, Model 2 further adjusted for lifestyle factors

## Discussion

This large-scale population-based study is one of the first studies to examine the associations between daily salt intake and health outcomes including obesity, diabetes and hypertension among adult population in Ningbo, China.

The current study indicates that mean daily salt intake among adults was extremely higher than Chinese DRI (6 g/d) [26] and WHO recommendation (5 g/d) [15]. The majority of our participants (84%) consumed more than the Chinese DRI level. Observational studies showed that the majority of Chinese adults had high salt consumption [17, 27, 28], which is in line with our findings. One recent report from China Health and Nutrition Survey in 2002 showed that Chinese national level of salt intake was 12 g/d, but the intake levels were diverse according to geography [29]. Our finding is slightly higher than the national level in 2002 as dietary behavior, food preparation and seafood in local area contributed to high amount of daily salt intake. Likewise, high salt intakes among adults were reported 18.0 g/d in Turkey measured with 24-h urine collections [30]; 10.9 g/d in India estimated with a three-day food diary [31]; 10.7 g/d in Portugal using 24-h urine collections [32]; 9.2 g/d in the USA with 24-h urine collections [33]; 11.5 g/d in Italy with FFQ [34] and 9.0 g/d in Australia using 24-h urine collections [35], although dietary assessments were different for collecting dietary information.

Our study showed that daily salt intake was the most strongly associated with higher odds of developing blood pressure status, which is consistent with several previous observational studies [36–38]. In contrast with blood pressure, obesity and fasting blood glucose were not significantly associated with daily salt intake. Results from the INTERMAP study in 1996–1999 involving 17 population samples aged 40–59 years showed that significantly adverse association of 24-h urinary dietary sodium excretion with blood pressure after with or without control for BMI [39]. Similarly, ALTURK study showed that salt intake together with gender, age and BMI was the significant predictors of both SBP and DBP [30]. The results from a worldwide epidemiologic study-INTERSALT study, conducted on dietary salt/sodium and hypertension with a 24-h urinary sodium, demonstrated the increased salt intake elevating blood pressure level with age [40]. The increase in salt sensitivity with age may result in a raise in salt intake. We found that senior generation had significantly higher daily salt intake compared to young adults in our study. Additionally, blood pressure of senior generation in the prehypertension and hypertension groups is more sensitive to daily salt intake [41, 42], although no significant difference in daily salt intakes was observed among obesity and diabetes status. Salt sensitivity could be genetic predisposition for Southeast Asian populations compared to Caucasians [43]. Therefore, senior Chinese generation is prone to develop prehypertension and hypertension due to salt sensitivity.

One conclusion from an epidemiological study indicated that processed foods as a strong risk factor for cardiovascular disease may cause an association of dietary salt with weight gain and obesity [41]. Excessive processed foods and dietary salt are related to passive overconsumption of energy, fat, cholesterol and fluid; may hereby contribute to weight gain [44]. Higher salt and energy intakes were reported among obese subjects [28], which is in contrast to our findings. No significant difference in daily salt intake was found among the status of BMI and fasting blood glucose. A supporting result from a Korean national health and nutrition examination survey indicated no association of sodium excretion with obesity and metabolic disorder in senior generation [41]. The possible reason is that those participants, who are categorized into obesity and diabetes groups, might be more careful about their dietary intake since they might have knowledge of weight gain, blood glucose, diabetes, and dietary instruction. A recent report from the UK National Diet and Nutrition Survey 2008/2009 to 2011/2012 indicated that a more 1-g/d salt intake was associated with 26% higher odds of overweight and obesity after adjusting the confounding factors, socio-demographic factors and total energy intake [45]. A high-salt diet may increase fasting ghrelin levels, thus, regulate appetite, glucose homeostatic, insulin resistance and fat accumulation [46], which most likely involves in the mechanism of obesity and diabetes. In addition, a high-salt diet rich in dietary Na^+^ density causes dietary K^+^ depletion, thus, results in negative impact on blood pressure regulation, and health benefits on cardiovascular diseases since high Na^+^ density may increase left ventricle wall and mass [47].

Moreover, poor nutrition and sedentary lifestyle are the best-known inducers developing chronic diseases. Besides nutrition and lifestyle, education is an essential factor as well causing chronic diseases. Knowledge and awareness related to social class are the factors of determination of dietary behavior and lifestyle [28, 48]. A high dietary sodium may be a reflection of poor diet quality and energy-dense diet. Interestingly, women consuming more than Chinese DRI was found to have almost 2 times higher prevalence of pre-hypertension than men did in our study. One recent study indicated that rural Chinese women had higher incidence of hypertension compared to urban ones due to less physical activity and health education [49]. A healthy dietary pattern high in fruit, vegetables and low fat diet led to lower blood pressure compared to a high salt and energy-dense dietary pattern [50]. Although overall daily salt intake was higher among rural participants in our study, daily salt intake of urban participants significantly increased among blood pressure status, hypertension group in particular. The dietary pattern and culture in the rural areas are still keeping a traditional Chinese way, while the dietary pattern in the urban areas is more diverse and closer to the Western dietary pattern such as fast foods and semi-prepared foods. This energy-dense dietary pattern changing the traditional Chinese dietary pattern and dietary culture has caused higher blood pressure, reported from a national level study in China [51]. Evidence proved that the majority of individuals are lack of knowledge of DRI for salt, and its relationship of salt with hypertension, less educated individuals in particular [28]. Less education and lack of knowledge resulted in participants consuming excessive daily salt intake, which is significantly associated with higher odds of hypertension.

### Strengths and limitations

The current study was the baseline study of a cohort survey, which was representative for the local Chinese citizens living in Ningbo, China. Additionally, it is the first study examining daily salt intake in relation to chronic diseases including obesity, diabetes and blood pressure in Ningbo. Biomarkers were reported by well-trained health workers.

Nonetheless, some limitations of this study need to be considered. First, causality cannot be inferred according to cross-sectional study design. Second, FFQ is not an accurate assessment collecting infrequently consumed foods. Then, total energy intake was not adjusted in the ordinal logistic regression model. Thus, it may influence associations of daily salt intake with health outcomes of chronic diseases. Moreover, information on dietary information relies on individuals’ memory and might be biased towards misreporting.

## Conclusion

Mean daily salt intake among the majority of participants exceeded Chinese DRI. Potential confounding and lifestyle factors were playing important roles in daily salt intake among risk groups, blood pressure in particular. Less educated and rural individuals, who were overweight/obese or had high fasting blood glucose/blood pressure, consumed significantly more salt than those counterparts did. Daily salt intake was significantly associated with higher odds of developing prehypertension and hypertension. Therefore, salt registration initiative is essential in the management, policy and strategy of salt content for preventing and controlling the epidemic prehypertension, hypertension and its related chronic diseases. The future study needs to investigate causality of prospective cohort study.

## Abbreviations

BMI: Body mass index
CDC: Center for Disease Control and Prevention
CI: Confidence interval
DBP: Diastolic blood pressure
DRI: Dietary reference intake
FFQ: Food frequency questionnaire
NCD: Non-communicable diseases
OR: Odds ratio
SBP: Systolic blood pressure
WHO: World health organization
